# Renewable energy communities or ecosystems: An analysis of selected cases

**DOI:** 10.1016/j.heliyon.2022.e12617

**Published:** 2022-12-26

**Authors:** Kankam O. Adu-Kankam, Luis M. Camarinha-Matos

**Affiliations:** a Nova University of Lisbon, School of Science and Technology and UNINOVA - CTS, Campus de Caparica, 2829-516 Monte de Caparica, Portugal; b School of Engineering, University of Energy and Natural Resources (UENR), P. O. Box 214, Sunyani, Ghana

**Keywords:** Collaborative networks, Energy ecosystems, Peer-to-peer energy exchange, Sharing economy, Renewable energy communities, Virtual power plants

## Abstract

The rapid proliferation of renewable energy communities/ecosystems is an indication of their potential contribution to the ongoing energy transition. A common characteristic of these ecosystems is their complex composition, which often involves the interaction of multiple actors. Currently, the notions of "networking", "collaboration", "coordination", and "cooperation", although having different meanings, are often loosely used to describe these interactions, which creates a sense of ambiguity and confusion. To better characterize the nature of interactions in current and emerging ecosystems, this article uses the systematic literature review method to analyse 34 emerging cases. The objective is threefold (a) to study the interactions and engagements between the involved actors, aiming at identifying elements of collaboration. (b) Identify the adopted technological enablers, and (c) ascertain how the composition and functions of these ecosystems compare to virtual power plants. The outcome revealed that the interactions between the members of these ecosystems can be described as cooperation and not necessarily as collaboration, except in a few cases. Regarding technological enablers, a vast panoply of technologies, such as IoT devices, smart meters, intelligent software agents, peer-to-peer networks, distributed ledger systems/blockchain technology (including smart contracts, blockchain as a platform service, and cryptocurrencies) were found. In comparison with virtual power plants, these ecosystems have similar composition, thus, having multiple actors, comprised of decentralized and heterogeneous technologies, and are formed by aggregating various distributed energy resources. They are also supported by ICT and are characterized by the simultaneous flow of information and energy.

## 1 Introduction

Electrical energy is crucial nowadays, and without it, contemporary societies cannot function adequately. This claim stems from the fact that critical sectors of modern society, which include industries, housing, communication, road infrastructure, education, health, and the financial sectors, depend largely on electrical energy. However, unlike past societies, the modern society, which is touted as the Fourth Industrial Revolution has heavily been dependent on fossil fuels energy sources. As published by “our world in data” [1], Fossil fuels have been the fundamental driver or enabler of the technological, social, economic and developmental progress of this current Industrial Revolution. In [2], Forbes claimed that fossil fuels account for 84% of global energy use, although this conventional energy source is known for its limited availability [3] and adverse impact on the environment [4], human health [5], and economic activities [6]. Moreover, the dominant literature suggests that severe exploitation of the Earth's resources to satisfy society's growing demands for energy is troubling, and has contributed to the ongoing climate change catastrophe, which poses a ruinous risk to the sustainability of the planet. Due to the many environmental concerns that are associated with fossil fuels, the need for safer, greener and more sustainable energy sources is imminent. Currently, researchers and policymakers have realized the potential benefits of transitioning from fossil fuel sources to renewable sources [7]. This is because, unlike fossil fuels, renewable energy is known to be inexhaustible [8] and can replenish itself in a relatively short period, thus helping to overcome the finite-supply problem associated with fossil fuels. In addition, renewable energy sources are cleaner, self-replenishing, environmentally friendly, and less harmful to human existence on planet Earth.

Regarding the transition from fossil fuels to renewable sources, Navigant [9] asserted that the future of energy would be cleaner, mobile, intelligent, and smarter as it would be dominated by renewable sources. Evidence of this claim is visible in the form of new and widespread renewable energy-related technologies and services that are currently emerging at the peripheries of the power grid. The expectation is that these advances could be the technological drivers or enablers of the transition agenda, which aims to transform the current power grid towards a "digitized," "decentralized," "decarbonized," "democratized" and “smart” power network [10]. The projections of Navigant, as well as the ongoing energy transition, are also affirmed by other researchers, such as [11] and [12], regarding the future of energy. Some of the advances claimed by Navigant include (a) widespread integration of distributed generation, (b) peer-to-peer energy trading, (c) renewable energy communities, and many more. Besides these claims, several cutting-edge, innovative, and complementary technologies, such as (a) artificial intelligence, (b) cloud computing, (c) the Internet of Things, (d) cyber-physical systems, and (e) blockchain technology are also emerging within the energy terrain. The integration of these diverse, innovative and complementary technologies into the renewable energy landscape is paving the way for new types of organizations, relationships, partnerships, business models, and services that are beginning to show within the energy space [11, 13, 14]. Other aspects of the transition, as mentioned by Gartner in [10], involve new roles that are played by autonomous actors, such as asset owners, system operators, and other economic entities, who have also evolved to become active participants and key players in the transition.

Among the plethora of approaches that have been suggested to help increase the penetration of renewable energy sources, in support of the transition, is the notion of Renewable Energy Communities (RECs) or energy exchange/sharing ecosystems, as described in [15]. These ecosystems are gradually becoming an integral part of the grid system due to their potential benefits [16]. According to the European Parliament and the Council of the European Union [5], RECs are based on free and voluntary participation. They are autonomous and managed by stakeholders. REC members can produce their own energy, which can be used locally, stored, sold, or shared with others in the framework of a community. According to the European Commission [17], at least 2 million people in the European Union are involved in more than 7700 RECs. These RECs have contributed nearly 7% of the Union's installed capacity, with an estimated total renewable capacity of no less than 6.3 GW. In terms of financial investment, some 2.6 billion Euros have been invested in RECs to date. Similarly, in the United Kingdom, there are over 5000 RECs that have contributed over 60MW of energy to the country's energy stock, with over 23 million Pounds contributed to community benefit funds for the benefit of local communities.

This notwithstanding, the number, types, composition, and characteristics of actors who are found to participate in these RECs have changed in recent years. The new actors are currently found to be constituted of several diverse autonomous and heterogeneous entities that can participate in the exchange of goods and services either “for-profit" or "non-profit" within these ecosystems. Usually, these actors are driven by a common interest or some objective that is compatible or common to the involved actors. These actors may constitute (a) consumers, (b) prosumers, (c) third-party energy service providers, (d) distribution service operators, (e) utility companies, (f) non-profit organizations, (g) financial institutions, (h) academic/research institutions, and (i) decentralized autonomous organizations, among many others. According to [18], when all of these autonomous and heterogenous entities work together in the context of a community, it could help uncover new ways to generate value, such as earning revenue that could help participants mitigate the rising cost of energy in these communities, as well as promote investment in renewable energy.

The rationale behind the sharing principles that underline these ecosystems, and their operation follows the current trends in the notion of the “sharing economy”, which is also aliased as (a) the collaborative economy, (b) collaborative consumption, (c) on-demand collaborative economy, (d) peer-to-peer (P2P) economy, (e) zero-marginal cost economy, and (f) crowd-based capitalism [14]. In [19], the authors shed more light on this economy and emphasized that its proliferation is the result of a technological phenomenon that has enabled the advancement of online platforms that promote user-generated content, sharing, and collaboration. These platforms function as intermediaries or marketplaces that enable the exchange or shared use of goods and services from peers, both for-profit and non-profit, through intermediation, matchmaking, and value-added services [20]. In the European Union H2020 Ps2Share research project [20], eight different definitions of the sharing economy are mentioned. For their relevance to this study, five of them are quoted below:

a. “*.......... peer to peer sharing or access to underutilized goods and services, which priorities utilization and accessibility over ownership.*”
b. “*..........group of online platforms facilitating peer-to-peer forms of economic activity.*”
c. “*…......the use of online marketplaces and social networking technologies to facilitate peer-to-peer sharing of resources (such as space, money, goods, skills, and services) between individuals, who may be both suppliers and consumers.*”
d. “*…the peer-to-peer-based activity of obtaining, giving, or sharing the access to goods and services, coordinated through community-based online services.*”
e. “*……...consumers granting each other temporary access to under-utilized physical assets (“idle capacity, possibly for money.”)*.

From the above definitions, it can be argued that products, services, and market relationships are changing radically in this new economy. Goods and services are currently moving away from centralized ownership to decentralized sharing, impacting businesses and their modus operandi in a very disruptive way. Juxtaposing the sharing economy with the REC concept, it is observed that community members who generate their energy locally from renewable sources, which are located in their homes, offices, and factories, can share or trade their surplus or excess with other community members in a localized market for-profit or non-profit. This fact, therefore, helps to situate the REC concept in the context of the sharing economy.

Following the above, we propose the objectives of this study, which is to conduct a case study on 34 selected emerging cases of energy-sharing ecosystems. Our objectives are threefold. First, to gain some insight into the nature of the interactions that exist between the various actors participating in these energy-sharing ecosystems, namely, to study the interactions and engagements that occur between the involved actors to ascertain whether the interactions that govern these sharing behaviours emerge out of (a) networking, (b) coordination (c) cooperation or (e) collaboration relationships. The motivation for this objective is gleaned from the fact that the ongoing energy transition is likely to create a future scenario where thousand or possibly, millions of interconnected actors, smart devices, and intelligent systems co-exist and do work together. For such a synergy to be efficient, beneficial, and reliable, the involved entities must cooperate in a trustful way that brings mutual benefit to all. As claimed by [21] collaboration is the process through which a group of entities enhance the capabilities of each other. The process involves the mutual engagement of the participants so that they can together, solve, a common problem using a collective approach. Furthermore, collaboration enables entities to be more competitive against other competing entities or groups. It can also increase the survivability of the group in times of turbulence. Therefore, knowing the nature of interactions that exist between actors within these ecosystems could provide a good base to explore possible avenues to strengthen or increase the survivability, resilience and competitiveness of these ecosystems using collaborative techniques. To help achieve this objective we adopted concepts and background knowledge from the domain of collaborative networks (CNs) to help analyze these cases.

Our second objective is based on the fact that these ecosystems thrive and continue to proliferate as long as they are able to provide participants with secure, fast, trustful, efficient, and transparent services. Therefore, understanding the trends and the types of technologies that are being used to support these ecosystems also form a relevant aspect of this work. The motivation for this aspect of the study is to determine the extent to which digitalization and the digital transformation are impacting the energy sector, particularly RECs. The anticipation is that knowledge about the kinds of digital technologies that are currently trending in this space could help provide a fair assessment of the level of penetration of these technologies within the mentioned energy environment.

Finally, we aim at exploring how the composition and functions of these ecosystems compare to the concept of Virtual Power Plants (VPPs). This is because, superficially, these two concepts appear to be similar in terms of their composition, organization, and behaviour. Again, this research objective is motivated by the fact that VPPs are extensively used to provide ancillary services to the power grid. According to [22] VPPs are constituted of networks of distributed energy resources such as photovoltaic systems, wind turbines, Combined Heat and Power (CHP) units etc. By aggregating all these resources, a VPP can deliver similar services and subsequently trade in the same energy markets just like large central power plants. Furthermore, VPPs can be used to stabilize the power grids and create the preconditions for the integration of renewable energy sources into the grid. Superficially, these two ecosystems appear to have a similar composition. However, in terms of their organization, performance, and objectives, the case is not always the same. Having an in-depth understanding of the deficiencies of RECs in comparison to VPPS could open a new research opportunity to investigate how to reconfigure RECs in a way that could enable them to perform functions that are similar to VPPs. From these backgrounds, the following research questions are therefore suggested to guide the study:

RQ-1: How can the interaction between members of the selected energy ecosystems be described?
RQ-2: What technological enablers and trends underlie the establishment, operation, and service provision of these ecosystems?
RQ-3: How do the characteristics and functions of virtual power plants (VPPs) compare to those of renewable energy ecosystems?

The remaining sections of the article are as follows: Section 2 focuses on discussing background knowledge and some base concepts. Section 3 is dedicated to the research methodology, followed by the "focus cases" in Section 4. Finally, Section 5 draws some conclusions and provides some recommendations for future work.

## 2 Background knowledge and concepts

This section introduces some base concepts, namely from the domain of collaborative networks (CNs). The section also gives an overview of one case study, which is intended as an illustration to help map out and further establish the complex synergies or interactions that take place between the different players in the studied energy ecosystems.

### 2.1 Collaborative networks

In the last 20 years, the field of collaborative networks (CN) has grown, which is a good sign for a society that is based on knowledge. The rapid evolution of this body of knowledge emanated from challenges faced by engineering systems, business entities, and the general society to participate in collaborative ventures. Collaboration is known to bring benefits to the involved entities as mentioned in [21]. From this domain of study, we borrow some concepts that are typically used to describe joint endeavours. We use these definitions as a guide to help us analyse the considered cases, especially in the context of RQ-1.

### 2.2 Networking

A concept that involves communication and information exchange for the mutual benefit of the participants. A simple example of networking is the case where a group of entities share information about their experience using a specific tool. All of them can benefit from the information made available/shared. However, there is no common goal or structure that influences the form and timing of individual contributions, and therefore there is no common generation of value [23].

*Key features of networking:* communication and information exchange for mutual benefit; no targeted generation of common value.

### 2.3 Coordination

In addition to the exchange of information, coordination also involves the alignment/alteration of activities to achieve more efficient results. Coordination, which is the “act of working together harmoniously”, is one of the main components of collaboration. An example of coordinated activities happens when it is beneficial for several heterogeneous entities to share some information and adjust the timing of their lobbying activities for a new product to maximize their impact. However, each entity may have a different goal and use different resources and methods to make an impact. Value, in this sense, is mainly created at the individual level [23, 24].

*Key features of coordination:* communication and information exchange for mutual benefit; coordinated activities; each entity might have a different goal and may use its own resources and methods; value is created at the individual level.

### 2.4 Cooperation

Cooperation involves not only the exchange of information and adjustment of activities but also the division of some (non-extensive) labour among the participants. In this case, the added value results from the addition of individual "components" of value generated by the various participants almost independently. Based on client-supplier relationships and predefined roles in the value chain. A traditional supply chain is an example of a cooperative system. Each participant performs their part of the work almost independently (although coordinated with others). There is, however, a common plan, which in most cases is not jointly defined but instead designed by a single entity, and which requires some limited level of co-working, at least at the points where the results of one partner are delivered to the next partner. However, their goals are compatible because their results can be added or composed in a value chain leading to an end-product or service [23, 24].

*Key features of cooperation*: Communication and information exchange for mutual benefit; coordinated activities; division of labour; common plan but not jointly defined; sharing resources for achieving compatible goals; aggregated value is a result of value generated by the various participants in a quasi-independent manner.

### 2.5 Collaboration

Collaboration is a process by which entities share information, resources, and responsibilities to jointly plan, implement, and evaluate a program of activities to achieve a common goal. This concept is derived from the Latin word "collaborare" which means “to work together”. It can be seen as a process of shared creation, thus a process by which a group of entities enhance each other's capabilities. It implies sharing risks, resources, responsibilities, and rewards, which can also give an outside observer the image of a collective identity if desired by the group. Collaboration involves the mutual engagement of participants in solving a problem together, which implies mutual trust and therefore requires time, effort, and dedication. With collaboration, it is much harder to realize how much each person has added to the creation of value [23, 24]. There must always be a common purpose (goal) for the collaboration. This purpose could be expressed as a joint goal or problem to be solved together. Parties' having their individual goals is not enough for collaboration.

The following are some characteristics of collaboration:

- Preconditions for collaboration:
  • Parties mutually agree to collaborate, which implies accepting to share.
  • Parties keep a model of each other's capabilities.
  • Parties share a goal and keep some common vision during the collaborative process.
  • Parties maintain a shared understanding of the problem at hand, which implies discussing the state of their progress (state awareness of each other).
- Generic steps for a collaboration process:
  • Identifying the parties and bringing them together.
  • Defining the scope of the collaboration and defining the desired outcome.
  • Defining the collaboration structure in terms of leadership, roles, responsibilities, ownership, communication means and processes, decision-making, resource access, scheduling, and milestones.
  • Defining policies. For example, handling disagreements and conflicts, accountability, reward, recognition, and ownership of generated assets.
  • Defining the evaluation and assessment measures, mechanism, and process.
  • Identifying risks and planning contingency measures.
  • Establishing commitment.
- Key features of collaboration:
  • Communication and information exchange for mutual benefit.
  • Coordinated activities.
  • Joint planning and implementation.
  • Mutual engagement for joint creation.
  • Sharing risk, resources, responsibilities, and rewards.
  • Having an image of joint identity.
  • Having common or compatible goals.
  • Individual contributions to value created are difficult to determine.

**Figure 1:**
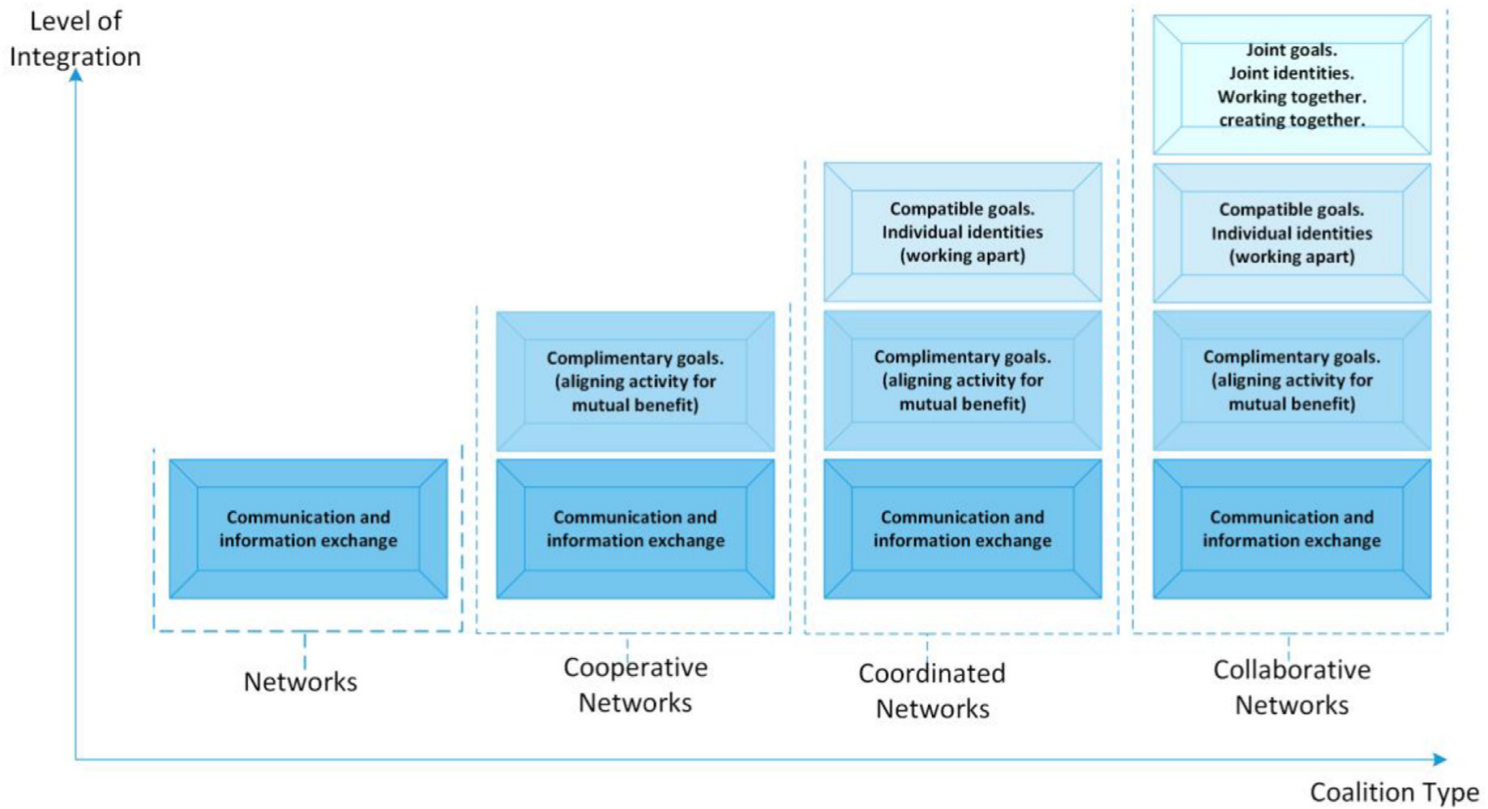
Classes of joint endeavours (adapted from [25]).

Figure 1 is an illustration of the types of joint ventures described above.

### 2.6 A panoramic view of interactions existing between actors in a sample of energy ecosystems

As mentioned at the beginning of this section, the result of a case study conducted in [15] is adopted here to shed further light on the assertion that these energy ecosystems are usually comprised of multiple, autonomous, and heterogeneous actors who happen to share a common environment and engage with each other in some form of interactions or relationships. From the many cases considered in the study, we borrowed the case of Feldheim [26], which is located in Germany, and shown in Table 1. This case is chosen because it has most of the traits that can be used to substantiate the claims that are being made. In Table 1, we illustrate these claims under six taxonomies: (a) key stakeholders of the ecosystem, (b) their key roles, (c) the kinds of energy resources that are owned by the ecosystem or its members, (d) the characteristics and relationships that exist between the roles, (e) the types of governance systems that have been implemented, and finally (f) the interaction of the community with the power grid.

**Table 1:** The Feldheim community showing the various actors, their roles, relationships, and governance type [15].

Main Actors/Key stakeholders	Roles			Governance structure	Interaction with the power grid
	Roles of Actors	Energy resources ownership	Characteristics & relationships between actors		
a) Feldheim New Energy Forum Foundation	i. Project financiers ii. Management body iii. Education and information	**Community-owned assets. These include:** 1. Wind turbine 2. Photovoltaic modules (PVs) 3. Biomass/Biogas 4. Combined heat and power (CHP) 5. Lithium-ion battery system	**I. Characteristics of roles**: Public-private partnership **II. Relationship between roles:** Distributed system with centralized management and hierarchical governance	A. **Governance type:** • Non-profit self-governance B. **Structure:** • Executive boards. • General assembly with voting rights. One member, one vote	Sends 90 % of the energy produced in the community to the grid. The community uses only 10%
b) Municipality of Treuenbrietzen	iv. Project financiers v. Project initiation and implementation				
c) Residents of Feldheim and Treuenbrietzen	vi. Project financiers vii. Centre of excellence viii. Energy consumers				
d) Feldheim Energiequelle GmbH	ix. Project initiation and implementation x. Project				
e) Farmers' Cooperative	xi. Project financiers xii. Landowners				

Referring to Table 1, it can be observed that some of the “main actors/key stakeholders” for this community are: (a) the Feldheim New Energy Forum Foundation, (b) the Municipality of Treuenbrietzen, (c) residents of Feldheim and Treuenbrietzen, (d) Feldheim Energiequelle GmbH, and (e) Farmers’ Cooperatives. Furthermore, focusing on the “Feldheim New Energy Forum Foundation” under the heading “main actors/key stakeholders,” it can be observed that the role of this stakeholder includes the following. Acting as (i) project financier, (ii) management body, and (iii) education and information provider.

In terms of energy resource ownership, it is found that all the energy assets located in the community are collectively owned by the community. The types of assets that are found are diverse and are constituted of (1) a wind turbine, (2) photovoltaic modules (PVs), (3) biomass/biogas, (4) Combined Heat and Power (CHP), and (5) lithium-ion battery storage system.

Similarly, the following outcomes are observed about the “characteristics and relationships between roles.” Two key descriptors are considered here: (I) the description of the “characteristics of roles” and (II) the description of the “relationships between roles.” Considering the characteristics between roles it is discovered that this is principally a public-private partnership. On the other hand, the “relationship between roles” is found to be a “distributed system with centralized management and hierarchical governance.”

Another key aspect of the case study is the governance structure that is observed in the case. The “governance type” as mentioned in subsection (A) of Table 1 is “non-profit self-governance.” Additionally, the “governance structure” as mentioned in subsection (B) is “executive boards” and a “general assembly with voting rights.” Thus, one man, one vote.

Finally, the interaction of the community with the power grid shows that the community consumes 10% of the locally generated energy and sends about 90% to the grid.

While this is a single example among many, the general observation for almost all the cases discussed in [15] seems to follow a similar or common trend in terms of composition, thus having multiple and autonomous actors, heterogeneity of actors, relationships between actors, and ownership of different kinds of energy resources. The types of ownership were wide-ranging, and the kinds of governance systems were also diverse.

## 3 Research method

In this study, the Systematic Literature Review (SLR) method is used to extract and analyse data from relevant works. SLR provides a rigorous and transparent method of literature review, assisting in the generation of robust and empirically derived answers to the focused research questions [27, 28]. In this section, we briefly discuss the different steps that were taken to select the articles that were used in the study.

### 3.1 Search criteria, cases identification and selection processes

**Figure 2:**
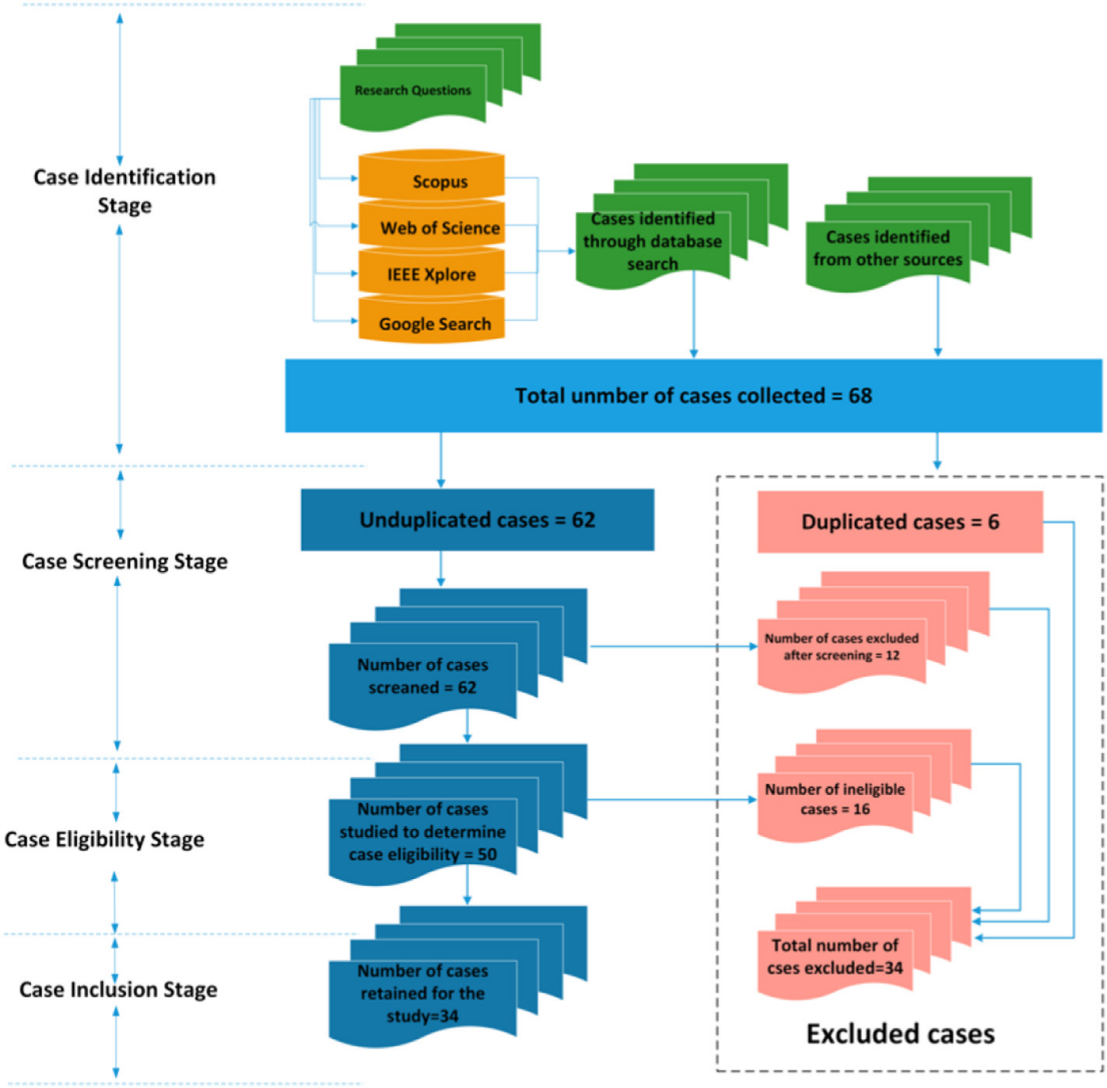
The flow of the systematic literature review process.

Here we describe the various stages through which energy community cases were identified and selected. The section describes the inclusion and exclusion criteria of these cases. Figure 2 and Table 2 are used to illustrate the various steps and processes that were used in selecting the cases. Cases that were considered are recent cases that were published between the years 2010 and 2020. The involved stages are as follows:

1 ***Case identification stage***: The first stage of the review process involves searching and gathering cases that are deemed relevant to the subject matter, in this case, the proposed research questions. The search is conducted from known databases of academic literature. The databases consulted included Scopus, Web of Science, IEEE Xplore, Google Scholar, Google Search, JSTOR, and Science Direct. Additional information is also gleaned from other sources, such as books and YouTube. Cases are selected from the search using the selection criteria described below. Articles that are initially collected from the search, which met the case selection criteria were sixty-eight cases. At this stage of the process, six cases were excluded due to duplication. The case selection criteria consisted of a set of keywords that were used in combination as shown in Table 2. Each keyword from column 1 is used in combination with all the keywords from columns 2, column 3, and column 4. For instance:
  • Renewable energy – Sharing – Online – Community.
  • Renewable energy – Sharing – Online- Ecosystem.
  • Renewable energy – Sharing – Online – Marketplace.
  • Distributed energy – Collaborations– Portal – Platform.
  • Distributed generation – Coalition – Portal– Platform.

**Table 2:** A combination of keywords that were used to search for cases from the search databases and other sources.

Column-1 Keywords describing the energy source	Column-2 Keywords describing the type of interaction	Column-3 Keywords describing the place of interaction	Columns-4 Keywords describing the type of ecosystem
Renewable energy Distributed energy Sustainable energy Green energy Distributed generation	Sharing Collaboration Cooperation Coordination Network Partnership Joint Co-evolution Coalition	Online Portal	Community Ecosystem Platform Portal Market place

For most of these cases, our primary or preferred source of information is the case's white paper, if it is available. This is because these documents provided detailed information about the related cases. Described below are the steps used for search criteria and case identification. These steps are further illustrated using Figure 2 below.

Case selection stage. Using Table 2 as a reference, four key components came together to determine a case's eligibility. These components are:

• *Description of energy source*: Under this item, the requirement for a case to be selected is that the energy within the ecosystem must be from a renewable source. The keywords, that were used included: renewable, distributed, sustainable, and green energy.
• *Types of interaction*: The type of interaction between the actors in the ecosystem is also of utmost importance. Our focus is on collaborative interactions. However, keywords such as sharing, cooperation, coordination, networks, partnership, joint, co-evolution, and coalition are considered for the search.
• *Place of interaction*: The place of interaction is expected to be described as online or a portal.
• *Description of the ecosystem:* Community, Platform, Portal, Marketplace, and Ecosystem are some of the keywords that are used to describe the expected ecosystems.

At the case identification stage, a total number of sixty-eight (68) potential cases are identified. Out of this number, six (6) cases are excluded for duplication.

2. Case screening stage: An overview analysis of the sixty-two cases that remained is conducted. At this stage, out-of-scope cases were identified and purged. A total of twelve (12) cases are screened out.
3. **case eligibility stage**: Eligible cases are cases that met the search criteria, fit the scope of the study and were not duplicates. These cases are retained for further analysis. At this stage, fifty (50) cases are considered. Further screening, involved a thorough study of each case to see if they meet the inclusion or exclusion criteria. At this stage of the process, a total of sixteen (16) cases are excluded for not meeting the inclusion criteria. All cases that did not meet the inclusion criteria are automatically excluded.
4. Case inclusion stage: In total, 34 cases are excluded, and 34 cases are retained for the analysis.

## 4 Results and discussion

### 4.1 Overview of the selected cases

During the process of search and selection process for the cases, it was found that the chosen cases came from many different types of businesses. The categories that are found include trading companies, financial/investment companies, solution developers, sole proprietorships, limited liability companies, ecosystems, platforms, and finally non-profit organizations (e.g., Prosume foundation). Although diverse, they all share some common characteristics that are relevant to the study. For instance, in almost all cases, the business entity brings together sellers and buyers on a single platform, portal, ecosystem, or community to facilitate the exchange of energy and related services that promote sustainable energy generation, consumption, and trading. In almost all cases, blockchain technology is used to facilitate secure and trustworthy transactions. Besides the fact that all these cases meet the inclusion criteria, their inclusion in the study also helps to widen the scope of the study by providing diversity in the cases considered. In addition, the cases were also found to be at different stages of development. Some cases are in the conceptual stage, others are in the developmental/prototype stages, and some are active/operational businesses.

In this section of the study, a detailed analysis of the 34 selected cases is performed. In Table 3, a brief description of each case and its core functions are tabulated.

**Table 3:** A Summary of the selected cases for the study.

Index	Cases	Description	Core functions
1	BittWatt	A smart and flexible system that makes the most of all energy sources and enables users to exceed their customer's needs in a balanced and financially efficient ecosystem that learns and evolves [29].	1. To provide blockchain-enabled peer-to-peer energy trading platforms and services. 2. A new marketplace for electricity balancing and trading
2	Electrify Network	A technology start-up that aims to bring the process of investing in renewable energy plants, smart micro-grid deployment, transactive energy marketplaces, and self-sustained smart city development to a grass-root level [30].	1. To build renewable power systems and produce exclusively green energy that will be sold to utility providers and government-owned entities. 2. To build a dynamic platform for peer-to-peer (P2P) energy trading using blockchain. 3. Contribute to the building of self-sustaining smart city pilot projects. 4. To provide venture capital for renewable energy projects.
3	Electrify.Asia	An ecosystem that allows consumers to buy their energy directly from peers or electricity retailers using the blockchain and smart contracts [31].	1. P2P energy trading platform. 2. Promote the use of "synergy" and "marketplace 2.0," which are web and mobile application interfaces that let people make smart contracts with their energy providers.
4	ElectronConnect	Provides marketplace infrastructure, in the form of a multi-market launch and hosting facility, that enables network operators, distributed energy resource operators, and others to interact and unlock market-based efficiencies [32].	1. A service provider that provides a marketplace or platform as a service
5	EnergiMine	A platform that matches customers with small generators. Aims at creating a global ecosystem where users are rewarded with tokens for energy-efficient behaviours. For example, if a commuter is encouraged to use public transport by the local city authorities, they could be rewarded by being given an ETK token [33].	1. A service provider with a platform that achieves two objectives: •P2P marketplace for energy trading over the blockchain. •Incentivization & reward scheme for energy-saving behaviours.
6	Energo Labs	Start-up with the intent of creating a P2P platform for a distributed energy system using blockchain technology with a particular focus on microgrids [34].	1. The company provides a platform that integrates blockchain technology into the energy sector to establish Decentralized Autonomous Energy (DAE) communities, enabling enterprises to convert energy into digital assets and use them in P2P, machine-to-machine, electric vehicle-to-virtual grid, green card trading, carbon trading, and virtual net electricity billing forms.
7	Energy Web Foundation	A low-carbon, customer-centric electricity system ​that enables ​any energy asset ​owned by ​any customer ​to participate in ​an energy market [35].	1. Peer-to-peer energy trading service provider.
8	EnergyNet	A secure and cloud-based software-as-a-service platform that solves the most crucial challenges of Transactive Energy. Thus compensation for providing distributed energy services via the distributed electric grid [36].	1. To provide a blockchain-enabled peer-to-peer energy trading platform.
9	Enosi Foundation	The Enosi Platform permits energy providers to offer less expensive community-based energy programs. Using the Enosi Protocol, households with solar energy systems can become prosumers and sell excess energy to buyers of their choosing at prices they determine. They will be able to engage in peer-to-peer trading and community-owned generation, as well as take advantage of offers from innovative new energy retailers that benefit from the Enosi Platform's lower cost structure. [37].	1. Leverages smart meter technology that digitizes energy data, and combines this data with recent advances in distributed ledger technology to deliver: •A more efficient and secure transactive services platform. •Access to competition throughout the value chain •The ability for distributed renewable generation to be accessed through community energy schemes and markets •Market-led adoption of decentralized energy solutions
10	EtainPower	A platform that creates a compelling new channel for global investors to access and invest in renewable energy projects [38].	1. To provide renewable energy financing and a trading platform that is empowered by both blockchain and AI technologies.
11	Greeneum	Blockchain-powered, sustainable, scalable, and secure energy and data trading platform [39].	1. Provide Software as a Service (SaaS) for grid operators and utilities 2. Premium services (obtain financial and performance reports, maintenance operation reports, and actionable insights that can potentially lead to higher profitability) for solar and green energy producers 3. Provide smart contract services 4. Advertisements: The platform will attract a high volume of repeat users, which will allow companies to advertise their products and services for a fee.
12	Grid+	The Grid + agent leverages AI to understand and predict consumers' energy usage habits. With real-time pricing data, the agent makes smart decisions on behalf of the purchaser of energy in the most cost-effective way with zero effort from users [40].	1. Develop cutting-edge secure hardware and software to enable the use of cryptocurrency. 2. Management of digital assets 3. Trading in energy.
13	GridX	This provides a financial operating system to enable utilities, retail energy suppliers, distributed energy resource providers, energy service providers, and their customers to operate and participate in decentralized energy markets [41].	1. Enable utilities, retail energy suppliers, third-party energy services providers, and their customers to operate and participate in decentralized energy economies.
14	Hive Power	A turnkey solution to create and manage local energy communities on the blockchain called the “Hives”, provides economic optimization for participants. A Hive is a distributed energy market platform regulated through smart contracts where every user can buy and sell electrical energy [42].	1. P2P energy trading platform service provider.
15	Jouliette (Spectral)	A blockchain-based platform that enables individuals and communities to manage and share renewable energy produced locally [43].	1. Selling renewable energy. 2. Purchasing renewable energy.
16	KWHCoin	A blockchain-based solution for a platform that makes it easy and cheap to add renewable energy and distributed energy resources to the grid [44].	1. A digital currency that uses a blockchain to turn information about distributed energy resources into digital tokens.
17	Lition	It is a P2P energy trading platform that connects renewable energy producers with smart consumers directly [45].	1. Aims to make green electricity simple and flexible by directly connecting producers and customers.
18	LO3 Energy/Brooklyn Microgrid	A blockchain-based platform that enables decentralized business models and innovative technologies related to energy [46].	1. A blockchain-based platform that enables decentralized business models and innovative technologies related to energy. 2. They operate the Exergy System.
19	NAD Grid	A P2P energy exchange platform that is backed by advanced blockchain technology. [47].	1. A decentralized P2P electricity trading platform. 2. It enables buyers and sellers to trade electricity using the Eden token (cryptocurrency).
20	Peer 2 Peer Energy Protocol (P2PEP)	A distributed energy trading application that lets both small and large clean energy producers and consumers connect over the blockchain [48].	1. A blockchain-enabled P2P energy trading service provider.
21	Power Ledger	A trustful, transparent, and interoperable energy trading platform that supports a growing number of energy applications [49].	1. A platform that can provide real-time metering data, collection of big data, the right to access and dispatch assets, rapid transaction settlement, and network load balancing. This is applied in areas such as: • P2P Trading. • Neo-retailing. • Microgrid/embedded network. • Wholesale market settlement. • Autonomous asset (AA) management. • -Distributed market management: • Electric vehicles charging. • Carbon trading. • Transmission exchange.
22	Prosume Foundation	A Swiss foundation and non-profit organization with a vision of empowering communities to exchange energy assets in a P2P fashion using a blockchain-based online market [50].	1. Aims to develop a platform that will be used by utility companies, grid operators, system integrators, and communities to easily build local ecosystems and online marketplaces.
23	Pylon Network	A blockchain network designed to create an open renewable energy exchange community that will provide signals and financial incentives to the energy markets [51].	1. To facilitate the growth of digital energy services, such as the transformation of the energy market to a consumer-centric and energy-as-a-service model, in the era of digitalization.
24	Share & Charge	A marketplace/community that provides solutions for electric vehicle charging. It enables simple, secure, and smart charging services based on the Open Charging Network (OCN) [52].	1. A B2B service provider that provides simple, secure, and smart charging services based on the OCN.
25	Solar Bankers	Aims to develop a global network of self-sufficient, decentralized renewable energy communities and, through digitization, which is enabled by the Solar Bankers coin, which may transform electricity into a globally exchangeable commodity [53].	1. An international renewable energy company that is focused on the development of a global network of self-sufficient, decentralized, renewable energy communities.
26	Solar IoT	A P2P blockchain energy grid, that allows individuals to buy and sell energy to others in a fully open marketplace on the Ethereum Blockchain, where prices are low and energy is abundant [54].	1. Financing of solar projects. 2. P2P energy trading service provider.
27	SonnenCommunity	A community where members (households) can store and use their self-generated energy using Sonnen's intelligent energy storage system, the SonnenBatterie. [55].	1. Develop and promote the use of the “sonnenBatterie”, an intelligent energy storage system. 2. Communalization of energy storage. 3. Community-based energy sharing. 4. Provide e-mobility services.
28	Sun Exchange	Sun Exchange enables people to locate their solar panels in the optimal places on the planet for the good of the owners, and the energy users, as well as offering an indirect benefit to the entire world population [56].	1. To provide a peer-to-peer solar leasing platform.
29	SunContract	A platform for trading energy that will create the SunContract energy pool and make it easier for people to buy and sell electricity directly from each other using blockchain technology [57].	1. A service provider that uses the SunContract pool for the sale and purchase of renewable energy.
30	Tarus Project	Using electric vehicles (EV) as a mobile power transmission network [58].	1. Blockchain-enabled peer-to-peer energy trading and EV charging services.
31	Toomuch.energy	Toomuch.energy transforms neighbourhoods into fully digital energy communities with a range of P2P services and market choices [59].	1. To provide a blockchain-enabled peer-to-peer energy trading platform.
32	Verv VLUX	Verv combines innovations in machine learning, blockchain, AI, IoT, and energy storage to help develop peer-to-peer energy trading using the Verv Trading Platform (VTP). The ecosystem has been designed to facilitate trading at the grid edge [60].	1. Peer-to-peer energy trading service provider. 2. Deployment of IoT devices such as the VHH to manage household energy consumption and data collection.
33	Volt Market	Volt Markets disintermediates traditional energy markets and enables monitoring, managing, and trading of energy and energy attributes in a P2P market on the Ethereum blockchain [61].	1. Providing a platform which is driven by smart contracts on the Ethereum blockchain. Volt Market says it will make a system that is more secure, clear, and efficient than the ones that are already in place
34	WePower	WePower is a blockchain-based green energy financing and trading platform. It connects energy buyers (households, investors, or market makers) directly with green energy producers to facilitate the upfront purchase of energy at below-market prices. It uses energy tokenization to standardize and simplify the currently existing energy investment ecosystem. It is claimed to provide access to live trade in renewable energy globally for everyone [62].	1. Financing green energy projects. 2. P2P energy trading service provider.

### 4.2 Summary and cases comparison

Table 4 below provides a summary of the cases, detailing how they compare to one another. Generally, all the cases can be categorized under two generic groups, namely: (a) Platform as a Service (PaaS) and (b) Software as a Service (SaaS). Under the PaaS category, we identified several types of sub-services. These include (i) IoT integration services (ii) renewable energy project financing services (iii) creating a marketplace for trading (iv) encouraging green behaviours (vi) compensation schemes (vii) providing energy storage services (viii) facilitating the integration of renewable energy sources into the grid (ix) offering charging services for electric vehicles (x) offering load balancing services and (xi) supporting the idea of a "smart city" The corresponding cases for each sub-service and their total numbers are also shown in the table. Similarly, under the category of SaaS, two key services are found. These are (i) software to provide reporting services (financial, performance, and maintenance operation reports). Other services found include advertisements that could generate additional income for the platform/ecosystem. (ii) the provision of software agents for decision-making services.

**Table 4:** A summary and case comparison.

Types of Services	Nature of service	Cases	Total number of cases
Platform as a Service	Facilitating IoT integration	Verv VLUX	1
	Financing of renewable energy projects	GridX, Solar Bankers, Solar IoT Sun Exchange, WePower, EtainPower, Electrify Network	7
	Facilitating a marketplace for trading	Electrify.Asia, ElectronConnect, EnergiMine, Enosi Foundation, EtainPower, Lition, LO3 Energy/Brooklyn Microgrid, NAD Grid, Peer 2 Peer Energy Protocol (P2PEP), Power Ledger, Prosume Foundation, Pylon Network, SunContract, Toomuch.energy, Verv VLUX Volt Market	18
	Promoting green behaviours	Energo Labs, Power Ledger	2
	Facilitating compensations	EnergyNet	1
	Facilitating energy storage	SonnenCommunity	1
	Facilitating integration of renewable energy in the grid	KWHCoin	1
	Facilitating electric vehicle charging services	Tarus Project	1
	Facilitating load balancing	BittWatt, Power Ledger	2
	Facilitating smart city concepts	Electrify Network	1
	Facilitating the management of energy asset and data	Energy Web Foundation, Greeneum, Power Ledger, Volt Market, Power Ledger	5
Software as a Service	Reporting and Advertisement	Greeneum	1

### 4.3 Addressing RQ-1: how can the interaction between members of the selected energy ecosystems be described?

The context of question “RQ-1” is to see if the interaction between the actors in these ecosystems can be characterized as either networking, coordination, cooperation, or collaboration. The outcome of this research question is summarized in Table 5. To help answer this question, we studied each case to determine the presence of some key features that are used to describe collaboration. Ten key features are used. For a case to be considered as engaging in collaboration all of the features of collaboration must be visible in the case. In cases where these features are partially visible, the cases were further analyzed to determine whether they could fit the description of networking, coordination, or cooperation. For this purpose, the following features are used:

1. *Communication and information exchange for mutual benefit*: Is there evidence of communication for mutual benefit?
2. *Coordinated activities*: Are the activities of these ecosystems coordinated?
3. *Joint planning and implementation*: Is there evidence of joint planning and joint implementation?
4. *A jointly defined common plan*: Is there evidence of a common plan that is jointly defined?
5. *Mutual engagement for joint creation*: Is there evidence of mutual engagement resulting in the joint creation of a product?
6. *Sharing risks, resources, responsibilities, and rewards*: Is there evidence of risk, resources, and rewards sharing?
7. *Having an image of joint identity*: Do members possess or assume a joint or collective identity?
8. *Having common or compatible goals*: Is there a common goal (or compatible goals) that is jointly agreed upon?
9. *Value co-creation*: Do members create value together?
10. *Division of labour*: Is there a division of labour?
  ⁃ Yes: means there is sufficient evidence to conclude.
  ⁃ Partial: means there is some evidence, but not concrete or sufficient to conclude.
  ⁃ No: means there is no evidence.

**Table 5:** Analysis of cases to determine the feature of Collaboration or Cooperation (RQ-1).

Index	Cases	Communication and information exchange for mutual benefit	coordinated activities	Joint planning and implementation	Common plan jointly defined	Mutual engagement for Joint creation	Sharing risk, resources, responsibilities, and rewards	Image of joint identity	Common goals	Value co-creation	Division of labour	Conclusion/Remarks
1	BittWatt	Partial	Partial	No	No	No	Partial	Yes	Yes	No	Yes	Cooperation
2	Electrify Network	Partial	Partial	No	No	No	Partial	Yes	Yes	No	Yes	Cooperation
3	Electrify.Asia	Partial	Partial	No	No	Partial	Partial	Yes	Yes	No	Yes	Cooperation
4	ElectronConnect	Partial	Partial	No	No	Partial	Partial	Yes	Yes	No	Yes	Cooperation
5	EnergiMine	Partial	Partial	No	No	Partial	Partial	Yes	Yes	No	Yes	Cooperation
6	Energo Labs	Partial	Partial	No	No	Partial	Partial	Yes	Yes	No	Yes	Cooperation
7	Energy Web Foundation	Partial	Partial	No	No	Partial	Partial	Yes	Yes	No	Yes	Cooperation
8	EnergyNet	Partial	Partial	No	No	Partial	Partial	Yes	Yes	No	Yes	Cooperation
9	Enosi Foundation	Partial	Partial	No	No	Partial	Partial	Yes	Yes	No	Yes	Cooperation
10	EtainPower	Partial	Partial	No	No	No	Partial	Yes	Yes	No	Yes	Cooperation
11	Greeneum	Partial	Partial	No	No	Partial	Partial	Yes	Yes	No	Yes	Cooperation
12	Grid+	Partial	Partial	No	No	Partial	Partial	Yes	Yes	No	Yes	Cooperation
13	GridX	Partial	Partial	No	No	Partial	Partial	Yes	Yes	No	Yes	Cooperation
14	Hive Power	Partial	Partial	No	No	Partial	Partial	Yes	Yes	No	Yes	Cooperation
15	Jouliette (Spectral)	Partial	Partial	No	No	Partial	Partial	Yes	Yes	No	Yes	Cooperation
16	KWHCoin	Partial	Partial	No	No	Partial	Partial	Yes	Yes	No	Yes	Cooperation
17	Lition	Partial	Partial	No	No	Partial	Partial	Yes	Yes	No	Yes	Cooperation
18	LO3 Energy/Brooklyn Microgrid	Partial	Partial	No	No	Partial	Partial	Yes	Yes	No	Yes	Cooperation
19	NAD Grid	Partial	Partial	No	No	Partial	Partial	Yes	Yes	No	Yes	Cooperation
20	Peer 2 Peer Energy Protocol (P2PEP)	Partial	Partial	No	No	Partial	Partial	Yes	Yes	No	Yes	Cooperation
21	Power Ledger	Partial	Partial	No	No	Partial	Partial	Yes	Yes	No	Yes	Cooperation
22	Prosume Foundation	Partial	Partial	No	No	Partial	Partial	Yes	Yes	No	Yes	Cooperation
23	Pylon Network	Partial	Partial	No	No	Partial	Partial	Yes	Yes	Aggregated value	Yes	Cooperation
24	Share & Charge	Partial	Partial	No	No	Partial	Partial	Yes	Yes	No	Yes	Cooperation
25	Solar Bankers	Partial	Partial	No	-	Partial	Partial	Yes	Yes		Yes	
26	Solar IoT	Partial	Partial	No	No	Partial	Partial	Yes	Yes	No	Yes	Cooperation
27	SonnenCommunity	Yes	Yes	Yes, using a self-learning software platform	Yes	Yes, using a self-learning software platform	Yes	Yes	Yes	Yes	Yes	Collaboration
28	Sun Exchange	Partial	Partial	No	No	Partial	Partial	Yes	Yes	No	Yes	Cooperation
29	SunContract	Partial	Partial	No	No	Partial	Partial	Yes	Yes	No	Yes	Cooperation
30	Tarus Project	Partial	Partial	No	No	No	Partial	Yes	Yes	No	Yes	Cooperation
31	Toomuch.energy	Partial	Partial	No	No	Partial	Partial	Yes	Yes	No	Yes	Cooperation
32	Verv VLUX	Partial	Partial	No	No	Partial	Partial	Yes	Yes	No	Yes	Cooperation
33	Volt Market	Partial	Partial	No	No	Partial	Partial	Yes	Yes	No	Yes	Cooperation
34	WePower	Partial	Partial	No	No	Partial	Partial	Yes	Yes	Aggregated value	Yes	Cooperation

**Summary of review outcome for RQ-1.** Based on the conducted analysis, the following observations can be made:

1 ***Communication and information exchange for mutual benefit****.* In all cases considered, there is some form of information exchange across the platforms for the benefit of participants. For instance, considering Power Ledger, the platform facilitates information sharing that enables participants to identify and select different clean energy sources according to their desired preferences. In addition, members have access to information that enables them to select and trade with their neighbours. Additional information that is shared for the benefit of participants involves an incentive program dubbed the "Green Energy Loyalty Rewards Program." Although there is evidence of information exchange, the objective of exchanging information is not aimed at solving a common problem or creating some value together. In other words, there is no common goal or agreed objectives, to which this communication is intended to contribute. Since the study found some level of communication and information exchange as well as some level of benefits, even though they are not mutual, we can say that the level of communication and information exchange is "partial" in almost all cases except SonnenCommunity. In this community, the households are installed with Sonnon intelligent batteries, which are managed locally using the Sonnon intelligent energy management software. This software collects information about the household's energy generation and consumption. This information is shared with the central Sonnon VPP software using the Sonnen digital networking platform as the communication channel. The VPP software collects similar information from all households in the community and uses this information to make decisions related to the provision of VPP services. The goal of sharing this information is common to all households, thus facilitating decision-making towards the provision of VPP services to the grid, which is a common goal for the community.
2 ***Coordinated activities****.* In all cases considered, there is some level of coordination between service providers and participants. There is also evidence of coordination among the participants. However, coordination among members of these ecosystems is not based on mutual or clearly defined common objectives or goals. Service providers often facilitate this coordination. From the perspective of collaborative networks, coordination is defined as the act of working together harmoniously to achieve a common goal. However, the intent behind the type of coordination observed in these cases is not aimed at a common or agreed-upon goal. The coordination found did not involve the alignment or altering of individual activities so that, mutually, more efficient results could be achieved collectively. The types of coordination found in these cases can best be described as administrative or managerial roles that are played by third-party entities or service providers. From our observation, activities that are carried out in these ecosystems are not coordinated with collaboration in mind. Thus, it might be fair to give a "partial" status to this feature in all the cases that are analyzed, except for the SonnenCommunity, which has clear evidence of coordinated activities that tend toward working together.
3 ***Common plan, joint planning, and implementation****.* In all but one of the cases considered, there is no evidence of a common plan as well as joint planning and implementation designed by mutual consent or agreement among the actors in these ecosystems. However, as an exception, joint planning and implementation can be seen only in the SonnenCommunity. For this ecosystem, joint planning and implementation are achieved using a self-learning software platform [63]. Planning and implementation, in almost all cases, are performed in a quasi-independent manner, without the involvement of other participants. Individuals such as prosumers, investors, and consumers can engage in their planning and implementation activities without consulting other actors in the ecosystem. Therefore, the status of this feature for all the other cases can be described as “No.”
4 ***Mutual engagement in joint creation****.* Mutual engagement in joint creation means that parties come together to engage one another to jointly create a product, or a service, or increase the value of a product or service. In the considered cases, there is evidence of mutual engagement in most cases to help achieve the objectives of the various actors. However, in the case of the SonnenCommunity, it is completely different. In this particular case, the community uses a self-learning software platform to engage members in a way that allows the community to act as a VPP, selling renewable energy to the grid to create value. In this particular case, mutual engagement is visible, although through a software application. In most cases, the evidence is partially visible in the sense that value creation is observed. However, the process of creating value is not born out of any form of mutual engagement. Nevertheless, the types of products and services that are found can be the result of individual efforts that produce the results, without any underlying mutual engagement. It can therefore be inferred that value, in most cases, is created in a quasi-independent manner, without a common plan, agreement, or purpose. Thus, we consider that in most of these cases, mutual engagement for joint creation is "partial".
5 ***Sharing risks, resources, responsibilities, and rewards****.* There is evidence of partial sharing of resources, risks, and rewards. For instance, in the case of WePower, small energy producers who use WePower services and who cannot reach the 1 MWh certificate, which is the minimum requirement to trade in the energy market, are grouped as a single entity to sell their energy to the market. The constituents of this entity share the benefits proportionally among themselves. However, no explicit mention is made of sharing of risks and responsibilities. Similarly, the Pylon Network offers tools to simplify shared ownership processes through transparent, safe, and real-time monitoring of assets. This includes the distribution of profits or costs associated with the co-owned assets. Details about risk sharing are not explicitly mentioned in these cases. Moreover, it is observed that there is a common goal, namely, to aggregate energy and sells it to the grid. However, this goal does not result from a mutual plan based on a common objective with clearly defined roles or responsibilities for each member. Although the sharing of rewards and resources is explicitly mentioned in the Pylon Networks, the sharing of risks and responsibility is not mentioned. Because of this, we consider that sharing risks, resources, rewards, and responsibilities in these ecosystems is usually "partial."
6 ***Having an image of collective identity****.* Evidence of the notion of joint image or identity is found in almost all the cases considered. Although there is no explicit mention of a joint or collective identity, we acknowledge that all these platforms have some identity. At the time of joining the community, members are conversant with the names and identities of these ecosystems and nevertheless chose to join them. Since members signed up for these platforms knowing what they are, what they stand for and how they worked, our opinion is that identity is implied. Therefore, in all cases considered, this feature is considered present.
7 ***Having common or compatible goals****.* It is further observed in almost all the studied cases that sellers, buyers, investors, and service providers have “quasi-common” goals. These goals are found in two layers. The first observed layer is a sustainability goal. This goal is common among sellers whose primary objective is to generate, consume, and trade excess renewable energy, although this goal is not jointly conceived. Buyers, in most cases, also have a similar sustainability goal, which is to purchase and consume energy from renewable sources rather than fossil fuel-based sources. The goal of third-party actors, in the same sense, is to invest in renewable energy by creating a marketplace where sustainable energy and related services can be exchanged. These activities of third-party actors will consequently promote "sustainable consumption," hence an implicit goal. The second layer goal is an economic one. The goal of sellers, in this sense, is to maximize revenue from their sales. The goal of buyers, on the other hand, is to minimize the cost of their purchases. The goal of third-party actors is to maximize revenue and minimize costs. In addition, it can also be argued that prospective actors in these ecosystems may often have prior knowledge of the aims, objectives, and possibly goals of these ecosystems before joining. Accepting the terms and conditions at the time of joining may constitute an implied acceptance and alignment of the goals. In a hypothetical sense, all these goals can be considered compatible, although implicit or tacit in their design. Since the focus of this aspect of the study is centred on identifying “common goals,” it may be reasonable to infer the existence of some form of common “sustainability” and “economic” goals, although it can be argued that these goals were not jointly defined. Therefore, it may be reasonable to generalize the conclusion about “common goals” as being “present.”
8 ***Value co-creation****.* This notion implies the involvement of the customer and local stakeholders in the process of collectively creating new products or services [7]. In the context of collaboration, it is usually not easy to clearly identify the amount of "added value" that each member has contributed. Subsequently, it is not easy to devise general schemes to distribute revenues and liabilities [7]. There are other complementary factors that influence the behaviour of a network and thus its ability to generate value. These factors include the scheme of incentives, trust relationships and management processes, ethical code, collaboration culture, contracts, and collaboration agreements. These are key elements in a value co-creation environment. In almost all cases studied, these key value-creation elements or factors are not explicitly discussed. For instance, in the “Prosume” ecosystem, it is mentioned that “consumers will choose their energy provider according to their needs, possibilities, and ethics”. Ethics, as mentioned in this sense, is not about value co-creation. Also, an ecosystem like “EnergiMine” mentioned incentives in its white paper, but this relates to behaviour change and not necessarily value creation. Although many of the factors that influence the behaviour of a network towards value creation, as stated in [7], are mentioned sporadically in several cases, they have no special connotation to value creation. Three exceptional cases were found. These are the “Pylon Network” SonnenCommunity and “WePower”. These cases make explicit mention of aggregated value, which can be synonymous with value co-creation. Except for these three cases, all other cases can be considered as not co-creating value.
9 ***Division of labour****.* Division of labour, according to [64], is the process of dividing a task or job into smaller, interconnected sub-tasks, thereby generating efficiency gains due to the positive effects of specialization. The available evidence from the studied cases suggests that each member plays specific and specialized roles to achieve individual objectives. For instance, prosumers play their respective roles as producers, while consumers also play their respective roles as consumers. Investors and service providers also play their respective roles accordingly. Although these actors are found to be playing their natural roles as independent business entities, it can further be argued that these roles are “implicit roles” in the sense that each actor is likely to have foreknowledge of their expected role before joining a community. Furthermore, these roles are highly specialized, and the ecosystem is able to achieve its objectives by aggregating the outcomes of each actor's role. As a result, it may be commendable to infer that there are some forms of “division of labour” in these cases. However, they are more implicit and implied. The conclusion for this element is therefore in the affirmative.

### 4.4 Addressing RQ-2: what technological enablers and trends underlie the establishment, operation, and service provision of these ecosystems?

The outcome of this research question is summarized in Table 6. To help answer RQ-2, we studied each case to identify the key technologies being deployed in these ecosystems. Nine key technologies are observed, as briefly explained below:

**Table 6:** Summary of technological enablers for energy ecosystems.

Index	Cases		Enabling Technology								References
		Artificial Intelligence/Machine Learning		Blockchain Technology					ICT Architecture		
		IoT devices/Smart meters	Intelligent agents	Distributed Application (DApps)	Type of trading tokens/cryptocurrency	Distributed ledger technology	Smart contracts	Blockchain as a service platform (BaaS)	Cloud-based platform	P2P Network topology	
1	BittWatt	Yes	No	Ethereum	BWT	Public Blockchain	Yes	Yes	No	Yes	[29]
2	Electrify Network	Yes	No	Ethereum	Eden Token	Public Blockchain	Yes	Yes	Yes	Ye	[30]
3	Electrify.Asia	Powerpod	No	Ethereum	ELEC Token	Public Blockchain	Yes	No	No	Yes	[31]
4	ElectronConnect	Yes	No	Ethereum	-	Public Blockchain	Yes	Yes	No	Yes	[32]
5	EnergiMine	Yes	No	Ethereum	ETK	Public Blockchain	Yes	Yes	No	Yes	[33]
6	Energo Labs	Yes [EME 1.0]	No	Qtum blockchain	Qtum,	Qtum blockchain	Yes	Yes	No	Yes	[34]
7	Energy Web Foundation	No	Yes (D3A)	Ethereum	Tobalaba	Public Blockchain	Yes	Yes	Yes	Yes	[35, 65]
8	EnergyNet	Yes	No	Ethereum	Fiat Currency, any crypto	Public Blockchain	Yes	Yes	Yes	Yes	[36]
9	Enosi Foundation	Yes	No	Ethereum	Joul	Public Blockchain Private Blockchain	Yes	Yes	No	Yes	[37, 67]
10	EtainPower	Yes	No	Ethereum	EPR Token	Public Blockchain	Yes	Yes		Yes	[38]
11	Greeneum	No	No	Ethereum	Green tokens	Public Blockchain	Yes	Yes	No	Yes	[39]
12	Grid+	Yes	Yes: Grid + Smart agent	Ethereum	BOLT	Public Blockchain	Yes	Yes	No	Future implementation	[40]
13	GridX	gridBox		Ethereum		Public Blockchain	Yes				[41]
14	Hive Power	Yes	No	Ethereum	HVT	Public Blockchain	Yes	Yes	No	Yes	[42]
15	Jouliette (Spectral)	Yes	No	Ethereum	Jouliette	Public Blockchain	Yes	-	No	Yes	[43]
16	KWHCoin	Yes	No	-	KWHCoin	Public Blockchain	Yes	Yes	No	No	[44]
17	Lition	Yes	No	Hyperledger Fabric, Ethereum	Lition tokens	Public Blockchain	Yes	Yes	no	Yes	[45]
18	LO3 Energy/Brooklyn Microgrid	Yes	No	Ethereum	XRG	Public Blockchain	Yes	Yes	No	Yes	[46]
19	NAD Grid		No	Ethereum	Eden Token	Public Blockchain	Yes	Yes	-	Yes	[47]
20	Peer 2 Peer Energy Protocol (P2PEP)	Yes			PED Token	Public Blockchain	Yes	Yes	No	Yes	[48]
21	Power Ledger	No	No	Ethereum	POWR Tokens & Sparkz	Public Blockchain	Yes	Yes	No	Yes	[49]
22	Prosume Foundation	Yes	No	Ethereum	PEF Token	Public Blockchain	Yes	Yes	No	Yes	[50]
23	Pylon Network	Klenergy Metron	No	Ethereum	Pylon-Coin	Public Blockchain	Yes	Yes	No	Yes	[51]
24	Share & Charge	Yes	No	Ethereum	No	Public Blockchain	Yes	Yes	No	Yes	[52]
25	Solar Bankers	Yes	No	SkyLedger	Skycoin	Public Blockchain	Yes	Yes	No	Yes	[53]
26	Solar IoT	Yes	No	Ethereum	SolCredit	Public Blockchain	Yes	Yes	No	Yes	[54]
27	SonnenCommunity	Yes	Self-learning software	Not mentioned	N/A	Not mentioned	N/A	N/A	Not mentioned	Yes	[55]
28	Sun Exchange	Yes	No	Ethereum	SUNEX	Public Blockchain	Yes	Yes	No	No	[45]
29	SunContract	No	No	Ethereum	SNC	Public Blockchain	Yes	Yes	No	Yes	[57]
30	Tarus Project	Yes	No	Ethereum	TORUS	Public Blockchain	Yes	Yes	No	Yes	[58]
31	Toomuch.energy	Yes	No	Ethereum	-	Public Blockchain	Yes	Yes	No	Yes	[59]
32	Verv VLUX	Yes	No	Ethereum	VLUX Token	Public Blockchain	Yes	Yes	Yes	Yes	[60]
33	Volt Market	Yes	No	Ethereum	RECs	Public Blockchain	Yes		No	Yes	[61]
34	WePower	No	No	Ethereum	WPR Token	Public Blockchain	Yes	Yes	No	Yes	[62]

**Artificial Intelligence/Machine Learning***.* This element of the study focused on how artificial intelligence and machine learning technologies are being used in the studied energy ecosystems. Two subsections are considered under this section. These are:

**1. The integration of smart devices, IoT devices, smart meters, as well as intelligent agents.**

• ***Smart devices, IoT devices and smart meters.***

Under this item, we identified 29 out of 34 cases that integrate smart meters or IoT devices into their operation. Some examples include cases such as Electrify. Asia, which uses the Powerpod in its networks. Powerpod is simply an IoT gateway for reading and relaying real-time energy data to a central management system of the Electrify. Asia network, allowing an algorithm to match and process settlements. A second example is the Pylon network, which relies on a generic smart meter. The device is called the Klenergy METRON, and is described by the network as any digital meter, sub-meter, or electric vehicle charger that can record energy data on the Pylon Network's blockchain. A third example is the gridbox from gridx, which serves as a gateway to its Xenon platform, which is the interface to all connected Distributed Energy Resources (DERs) and is used for data collection and management of DERs. The Verv vlux network also introduced its core IoT product called the Verv Home Hub, which is a patented, self-installed energy hub that samples a home's electricity consumption approximately 5 million times faster than a smart meter. It uses machine learning algorithms to derive a real-time profile for key household appliances, providing homeowners with a view of their electrical appliances' current status [60].

• ***Intelligent Agents***.

As an example of the use of software agents, Energy Web Foundation - Decentralized Autonomous Area Agent (D3A) is an intelligent software agent that performs grid communication and control functions for physical assets. D3A allows any energy-consuming or energy-producing device to interact with other devices in a trustless blockchain environment, helping to optimize operational decisions locally and based on user preferences and system conditions [65]. Another intelligent agent application is found in the case of Grid+. In this example, whenever a customer signs up for Grid+, he will purchase a Smart Agent and buy BOLT (a cryptocurrency) from the Grid + web console. The Grid + smart agent, once registered, will allow customers to transfer BOLT tokens to the Smart Agent to pay for electricity in real time. An automatic payment option can be set up so that it can be refilled automatically if a Smart Agent runs out of BOLT. The Smart Agent will make digital signatures from a secure hardware enclave and act autonomously while still registered with Grid+ and owned by the customer [66].

**2.Blockchain Technology.**

In [68], a blockchain is described as a distributed and immutable ledger that facilitates the recording of transactions and the tracking of assets within a business network. An asset can be either tangible (such as a house, automobile, money, or land) or intangible (intellectual property, patents, copyrights, branding etc.). In a blockchain network, almost anything of value may be recorded and sold, lowering risk and expense for all involved. In the context of renewable energy communities, blockchain can give consumers greater control over their energy sources. Additionally, an immutable ledger provides secure and real-time updates of energy usage data and monitors trading between sellers and buyers. The transparency of public blockchains further reduces the chances of monetary or data exploitation [69]. Concerning the type of distributed ledger technology that is mainly used, it is found that all 34 cases studied used the public blockchain except one (the Enosi platform) that used a private blockchain. In this case, the private blockchain allows most computations to be validated by it rather than by the public chain. Another case (Energo Labs) also uses the Qtum blockchain. The use of blockchain technology in these emerging energy ecosystems is quite extensive and the following facets are worth mentioning:

• ***Distributed Applications (DApps).***

Ethereum, launched in 2015, is an open-source, blockchain-based decentralized software platform that can simultaneously integrate a cryptocurrency. Ethereum helps deploy Smart Contracts and DApps to be developed and executed without fraud, control, or interference from third parties. Ethereum offers both a platform and a programming language that runs on a blockchain and allows developers to build and publish distributed applications. 29 out of the 34 studied cases use the Ethereum platform to deploy their distributed applications. The use of other blockchain variants, such as Hyperledger Fabric, Qtum, and Skyledger blockchain, is also found in cases such as Lition, Energo Labs, and solar bankers, respectively.

• ***Trading Tokens (cryptocurrency).***

Of the 34 cases that are considered, 29 use cryptocurrencies as tokens for trade. These cryptocurrencies differ from ecosystem to ecosystem, and their values also vary. They are used as the main medium of exchange in these ecosystems instead of fiat currency in the real world. Many cryptocurrencies are supported on decentralized networks based on blockchain technology. One defining feature of cryptocurrencies is that they are generally not issued by any central authority, such as banks, making them theoretically immune to government interference or manipulation. For instance, in some cases, like "WePower," cryptocurrencies are used to tokenize energy. Tokenization of energy is a contracting scheme that is established between an energy producer and an energy buyer.

• ***Smart Contracts.***

Smart contracts (SC) are simply programs stored on a blockchain that run when predetermined conditions are met. SCs are computer protocols that facilitate, verify, or enforce the execution of a contract, thus making the need for a contract clause unnecessary. SCs often imitate the logic of contract clauses. SCs can support the exchange of money, property, shares, or anything of value in a transparent and conflict-free manner, avoiding the services of a middleman. Normally, a process would require payment to a middleman, a government agency, a bank, a lawyer, or a notary, and then a processing time before receiving goods or services. However, with smart contract technology, all these processes can be automated. In the studied energy ecosystems, information about transactions and arbitrations between sellers and buyers is achieved using smart contracts. All the cases considered use this technology.

**3. Other ICT Software Architectures.**

• ***Cloud-based platforms.***

Three cases are found to use cloud-based platforms. These are (a) Energy Web Foundation, (b) Verv VLUX, and (c) Electrify Network. For instance, Verv VLUX uses a decentralized cloud storage service to host the platform and related applications. Furthermore, the Verv Household Hub is designed to connect to the cloud via a Wi-Fi network and provide users with a central hub to control other cloud-connected household smart appliances [60]. Again, considering the Electrify Network, the platform sought to combine the reliability and robustness of the microgrids with intelligent, multi-tasking smart meters. This is made easier by software that runs in the cloud and makes all transactions and exchanges smooth, safe, and easy [30].

• ***P2P network technology.***

In all the cases considered, the platforms utilize the P2P network topology except in two cases, which are KWHCoin and Sun Exchange.

• ***Blockchain as a Service Platform (BaaS).***

BaaS is a relatively new development in the growing field of blockchain technology. For BaaS, a third-party service provider is responsible for setting up all the necessary blockchain technology and corresponding infrastructure for a fee. Once created, the provider continues to handle the complex back-end operations on behalf of the client. In almost all cases considered, third-party service providers are responsible for the blockchain infrastructure as a service offered to energy sellers and buyers.

### 4.5 Addressing RQ-3: how do the characteristics and functions of virtual power plants (VPPs) compare to renewable energy communities/ecosystems?

A VPP, according to [5], is a virtual entity with numerous stakeholders and decentralized multi-site heterogeneous technologies composed of dispatchable and non-dispatchable distributed energy sources and energy storage systems as well as electric cars and controllable loads. The use of information and communication technologies enables VPPs to act as the equivalent of a single power plant with the ability to manage and coordinate its operations, ensuring power and information flow among its stakeholders to reduce generation costs, maximize profits, and improve participation in demand response programs as well as trade within electricity markets. By juxtaposing the VPP concepts with RECs it can be found that both ecosystems are similar in a way. For instance, prosumers in RECs can aggregate their surplus or unused energy from the community and sell it to the grid. This enables RECs to also behave like VPPs.

VPPs can therefore be described as having the following features:

1. Is composed of multiple stakeholders/actors.
2. Is comprised of decentralized multi-site heterogeneous technology/systems.
3. Is formed by aggregating distributed energy resources.
4. Is supported by ICT.
5. Is characterized by the simultaneous flow of information and energy.
6. Can function like a single power plant

In this section of the study, the objective is to establish or ascertain how each of the 34 cases compares to a VPP. The focus here is on their characteristics and functions. As described above, items one (1) to five (5) represent the characteristics of a VPP while item six (6) focuses on its functions. To help explore these cases, the characteristics of the VPP are described above, thus, items 1–5 are framed into sub-research questions.

After a careful analysis, it is found that the answers to these sub-questions are generally in the affirmative, thus a “Yes” for all the cases. Further explanation is, however, given below:

Sub-RQ-A: Are these ecosystems composed of multiple stakeholders?

Yes. In all considered cases, these ecosystems are also found to be comprised of multiple stakeholders or actors. These stakeholders include prosumers, consumers, service providers, investors, communities, non-profit organizations, platforms, ecosystems, and many others.

Sub-RQ-B: Are these ecosystems comprised of decentralized multi-site heterogeneous technology?
  Yes. Distributed energy resources in these ecosystems are decentralized and located at multiple sites. There is evidence of heterogeneous technology use. RQ-2 provides an additional explanation of the types of technologies that are currently being used.
Sub-RQ-C: Are these ecosystems formed by aggregating distributed energy resources?
  Yes. Some cases demonstrate the aggregation of distributed energy resources, although not many of them.
Sub-RQ-D: Are these ecosystems supported by ICT?
  Yes. The studied ecosystems thrived with the support of computer networks. This is seen in all the cases studied. Community members are connected using an online portal.
Sub-RQ-D: Are these ecosystems characterized by a simultaneous flow of information and energy?
  Yes. There is information flow between community members and power flow from suppliers to consumers. All of this happens simultaneously.

In the context of the function, item 6 is also framed into a sub-research question as follows:

Sub-RQ-E: Do these ecosystems function like a virtual power plant, thus acting like a single power plant?

The outcome of this sub-research question is provided in Table 7 below.

**Table 7:** The outcome of sub-RQ-E.

Index	Cases	Do the ecosystems function like a virtual power plant, thus acting like a single power plant?
1	BittWatt	**No.** Reason: Does not appear to focus on the aggregation of Distributed Energy Resources (DERs), which is a key function that can enable these ecosystems to aggregate different energy generation units, which could result in the creation of some capacity that could enable them to act like a single power plant or a VPP
2	Electrify Network	**No.** Reason: Does not appear to focus on the aggregation of DERs.
3	Electrify.Asia	**No.** Reason: The focus is on the development and deployment of the “PowerPod” IoT device and P2P energy trading using the synergy and marketplace 2.0 applications
4	ElectronConnect	**No,** Reason: Focused on energy trading activities mainly
5	EnergiMine	**Partial.** Reason: Currently working with Elexon, a UK-based company to allow future aggregation of energy storage devices that can be traded on the platform to help balance the grid. Grid balancing is also one of the features of technical VPPs. Partial because this is yet to be implemented
6	Energo Labs	**No.** Reason: Does not appear to focus on the aggregation of DERs
7	Energy Web Foundation	No. Reason: Does not appear to focus on the aggregation of DERs
8	EnergyNet	**No.** Reason: Does not appear to focus on the aggregation of DERs
9	Enosi Foundation	**No.** Reason: The vertical hierarchical architecture of the Enosi network where consumers can only access the energy market through a Neo Retailer and subsequently a licensed retail supplier appears to limit the possibility of aggregating DERs to enable them to behave like VPPs.
10	EtainPower	**No.** Reason: Focusses on renewable energy project financing and not the aggregation of DERs
11	Greeneum	**No.** Reasons: Does not have the capacity to aggregate DERs.
12	Grid+	**No.** Reason: Act mainly as an energy trading platform with a focus on the deployment of the Grid + Smart agents. No emphasis is placed on the aggregation of DERs
13	GridX	**No.** Reason: Does not appear to focus on the aggregation of DERs
14	Hive Power	**Yes.** Reason: Based on the notion of “Self-Consumption Communities” (SCC) the community can sell excess solar power to the national grid e.g., during summer days and receive financial remuneration. This action replicates the behaviour of a VPP
15	Jouliette (Spectral)	**Partial.** Reason: The “Spectral Energy Control System” enables the seamless integration and control of energy storage devices, wind farms, PV plants, heat pumps, generators, and a wide range of other energy systems. This feature of the ecosystem can enable it to function as a VPP although this is not explicitly stated as a function of the ecosystem.
16	KWHCoin	**No.** Reason: Current focus is to promote the KWHCoin as a digital currency for energy trading
17	Lition	**No.** Reason: Does not appear to focus on the aggregation of DERs.
18	LO3 Energy/Brooklyn Microgrid	Partial. Reason: Have the capacity to support the local community in emergencies when the grid fails. -Has the potential to act as a single power source in future development
19	NAD Grid	**No.** Reason: Act mainly as an energy trading platform
20	Peer-2-Peer Energy Protocol (P2PEP)	**No.** Reason: Does not appear to focus on the aggregation of DERs
21	Power Ledger	**No.** Reason: The platform appears to focus on data collection, market management and/or pricing mechanisms
22	Prosume Foundation	**Yes.** Reason: One of the features of the platform is to facilitate the integration of Power-Plants and Micro-Grid management on the ESCO (Energy Sharing Company) model. This feature can enable the ecosystem to act like a VPP.
23	Pylon Network	**No.** Reason: The platform does not seem to support the aggregation of DERs to form the similitude of VPP although it is mentioned that stand-alone producers can sell surplus energy to the grid, there is no indication that this is achieved through aggregation of DERs.
24	Share & Charge	**No.** Reasons: The current focus is on EV charging. Does not have the capacity to aggregate DERs
25	Solar Bankers	**No.** Reason: Does not appear to focus on the aggregation of DERs.
36	Solar IoT	**No.** Reason: Does not appear to focus on the aggregation of DERs
27	SonnenCommunity	**Yes** Reason: The Sonnen Virtual Power Plant achieves this through its digitally networked swarm of home storage systems. If the electricity demand is higher than expected the batteries feed electricity into the grid. In the event of an unexpectedly high level of electricity production, they absorb the excess electricity from the grid.
28	Sun Exchange	**No.** Reason: Does not appear to focus on the aggregation of DERs
29	SunContract	**No.** Reason: Does not appear to focus on the aggregation of DERs
30	Tarus Project	**No.** Reason: Does not appear to focus on the aggregation of DERs
31	Toomuch.energy	**No.** Reason: Does not appear to focus on the aggregation of DERs
32	Verv VLUX	**No.** Reason: The focus is on the development and deployment of the Verv IoT devices and P2P energy trading using the Verv Trading Platform (VTP).
33	Volt Market	**No.** Reason: Does not appear to focus on the aggregation of DERs
34	WePower	**Yes.** Reason: small energy producers using WePower services that cannot reach the 1 MWh certificate, which is the minimum requirement for them to trade in the energy market, are pooled together as a single entity to sell their energy to the market.

## 5 Summary and conclusion of research findings

### 5.1 The existence of the potential to act like VPPs

The study reveals that most of these cases have access to diverse and vast numbers of DERs that are found to be connected in these networks. Incidentally, these DERs constitute a key asset for all VPPs. This, therefore, suggests that most of these ecosystems have access to the primary assets that could enable them to perform functions that may be similar to those of VPPs. Yet, in several of the studied cases, the ecosystems did not integrate features that could afford them the capacity to aggregate connected DERs. The focus of these ecosystems is predominantly on P2P exchanges.

### 5.2 Limited use of collaboration and related concepts

Another significant finding is that most cases operate at the level of cooperation rather than collaboration. In such cases, it can be suggested that collaboration could be a plausible mechanism that would allow actors in these ecosystems to come together, devise a common goal, and engage in collective actions that could result in the aggregation of outputs from connected DERs so that they could perform some function similar to a VPP. Knowledge, concepts, principles, and mechanisms from the domain of collaborative networks could be useful to adopt. Future research direction could focus on this area.

### 5.3 Limited use of intelligent agents

The types of enabling technologies that are found, appear to be driving the energy industry towards energy cloud 4.0 [9]. Some of the key highlights of these enabling technologies include the integration of AI, smart IoT devices, smart software agents, blockchain technology and related smart contracts, cryptocurrencies, and cloud-based applications such as platforms-as-a-service, etc. The direction of these ecosystems is towards greater sustainability, flexibility, autonomy, individualization, digitalization, and virtualization [9]. It is realized that these developments may introduce some complexities in terms of choices, decision-making, and preferences that may seem overwhelming and cumbersome for human users to single-headedly adopt and use. The need for some form of autonomous and complementary decision-making assistance to help navigate this myriad of options for optimized choices and decisions could be useful. However, the study reveals that all but two cases did not consider the integration of software agents into their ecosystems, although this concept has been shown to have good prospects in the implementation of complementary decision-making entities.

### 5.4 Cross-platform trading and interoperability between cryptocurrencies

Another noteworthy observation is the lack of evidence of cross-platform trading and interoperability between cryptocurrencies. Furthermore, unlike fiat currency, which can be converted from one currency to another, evidence of interoperability between the various cryptocurrencies is also lacking. With such limitations, the actors in these ecosystems are restricted in terms of choices and access to diversity in terms of affiliation, diverse energy sources, and related services.

## 6 Conclusion and future work

The objectives of this work are in three folds. These are (a) to characterize the interaction that exists between the actors in the studied ecosystems. (b) To extricate the technological enablers that are driving the existence and proliferation of these ecosystems and (c) to compare the characteristics and functions of a VPP to those of the renewable energy ecosystem cases. The result for the first objective is shown in Table 5. The table reveals that in almost all the considered cases, the interactions between the actors could be described as cooperation, except in one case. The result for the second objective is also summarized in Table 6. It can be observed from the table that the dominant technological enablers include IoT devices, smart meters, intelligent software agents, peer-to-peer networks, and distributed ledger/blockchain technology (including smart contracts, Blockchain as a Platform service, and cryptocurrencies). Other technologies that are found include Platform as a Service and Software as a Service. The result for the third objective is also shown in Table 7. It reveals that, in terms of characteristics, these two concepts are similar. This is because they are both found to be composed of multiple stakeholders or actors. They are comprised of decentralized multi-site heterogeneous technologies, systems, or assets and are formed by aggregating distributed energy resources. They are also found to be supported by ICT and are characterized by the simultaneous flow of information and energy. Although it has been shown that the characteristics of these energy ecosystems are similar to those of a VPP, they do not function in the same way.

In terms of their functions, the majority of energy ecosystem cases did not focus on energy resource aggregation, which is a key function that can enable these ecosystems to aggregate different energy generation units, which could result in the creation of some capacity that could enable them to act like a single power plant or a VPP. Since this functionality is generally absent, the majority of these ecosystems could not perform functions that could make them act like VPPs. However, there are four (4) cases that are found to possess the aggregation of the generation unit's functionality, and therefore these ecosystems could act like a single power plant or a VPP. These cases included Hive Power, Prosume Foundation, SonnonCommunity, and WePower. Other cases, such as EnergiMine and Jouliette (Spectral), are also found to show potential to function as a VPP.

The significance of this study can be seen from three perspectives. These are (a) the study reveals a research gap in terms of how actors of these ecosystems interact. The research opportunity in this direction includes the consideration of collaborative behaviours among members of these ecosystems. (b) The study has also helped to unveil the level of penetration of digital technology and digitalization within these energy ecosystems and finally, (c) It has helped to uncover the limitations of these renewable energy ecosystems and how these limitations could be overcome to enable them to perform other sustainability function comparable to VPPs.

In subsequent studies, we would like to know how the performance of these energy ecosystems could be improved if the interaction between members is reconfigured to allow them to collaborate rather than cooperate. Considering the claimed benefits of collaboration, as suggested by Camarinha-Matos and Afsarmanesh in [23], it is hoped that these ecosystems could yield better outcomes if the interactions between members are encouraged to take a collaborative form.

## Declarations

### Author contribution statement

All authors listed have significantly contributed to the development and the writing of this article.

### Funding statement

This work was supported by Project CESME (Collaborative & Evolvable Smart Manufacturing Ecosystem) and the Portuguese FCT program UIDB/00066/2020.

### Data availability statement

No data was used for the research described in the article.

### Declaration of interests statement

The authors declare no conflict of interest.

### Additional information

No additional information is available for this paper.
